# *QuickStats:* Age-Adjusted Percentage* of Adults Who Had Ever Used an E-cigarette,^†^ by Race and Ethnicity — National Health Interview Survey, United States, 2014 and 2018^§^

**DOI:** 10.15585/mmwr.mm6847a4

**Published:** 2019-11-29

**Figure:**
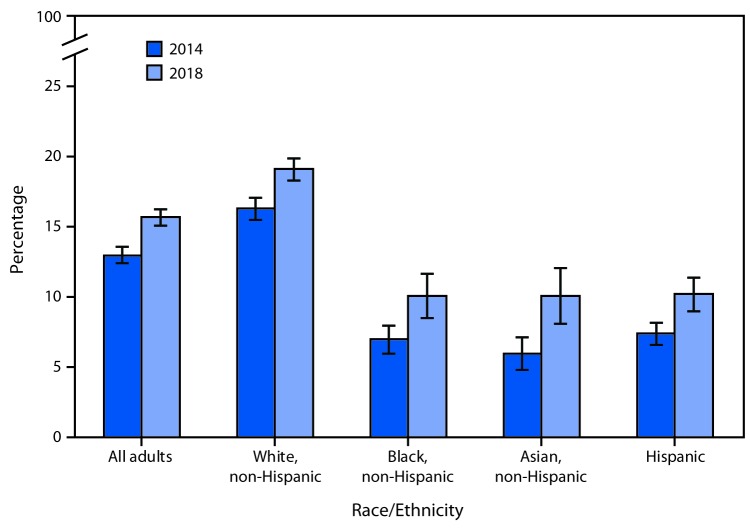
From 2014 to 2018, the percentage of all U.S. adults aged ≥18 years who had ever used an e-cigarette increased from 13.0% to 15.7% overall and, by race/ethnicity, increased among non-Hispanic white, non-Hispanic black, non-Hispanic Asian, and Hispanic adults. Non-Hispanic white adults were the most likely, in both years, to have ever used an e-cigarette. In 2018, 19.1% of non-Hispanic white adults had ever used an e-cigarette, compared with 10.1% of non-Hispanic blacks and non-Hispanic Asians and 10.2% of Hispanics.

**Source: **National Center for Health Statistics, National Health Interview Survey, 2014 and 2018 data. https://www.cdc.gov/nchs/nhis.htm.

